# Adipocyte miR-200b/a/429 ablation in mice leads to high-fat-diet-induced obesity

**DOI:** 10.18632/oncotarget.12080

**Published:** 2016-09-16

**Authors:** Cong Tao, Hongyan Ren, Pan Xu, Jia Cheng, Sujuan Huang, Rong Zhou, Yulian Mu, Shulin Yang, Desheng Qi, Yanfang Wang, Kui Li

**Affiliations:** ^1^ State Key Laboratory of Animal Nutrition, Institute of Animal Science, Chinese Academy of Agricultural Sciences, Beijing, China; ^2^ College of Animal Science and Technology, Huazhong Agricultural University, Wuhan, China; ^3^ Key Laboratory of Animal Embryo & Molecular Breeding of Hubei Province, Institute of Veterinary and Animal Science, Hubei Academy of Agricultural Science, Wuhan, China

**Keywords:** miR-200b/a/429, insulin resistance, high-fat-diet, knockout, adipose tissue, Pathology Section

## Abstract

Growing evidence demonstrates the important role of microRNAs (miRs) in regulating adipogenesis, obesity and insulin resistance. The miR-200b/a/429 cluster has been functionally characterized in mammalian reproduction; however, the potential role of the *miR-200 family in* adipocytes is poorly understood. The aim of our study was to investigate the physiological function of miR-200b/a/429 in the regulation of whole-body metabolism in terms of the activities and targets of this cluster in adipocytes. We generated adipocyte-specific miR-200b/a/429 knockout (ASKO) mice using a Cre-loxP system in which Cre expression was driven by the aP2 promoter. The ASKO and wild type (WT) littermate were fed a chow diet (CD) or high-fat-diet (HFD), and changes in body composition, metabolic parameters, energy homeostasis, glucose tolerance and insulin sensitivity were analyzed. The miR-200b/a/429 putative target genes were predicted and validated via luciferase reporter assays. We found that the HFD-fed ASKO mice gradually gained more body weight than the WT mice due to the increased adiposity. Decreased glucose tolerance and insulin sensitivity were also observed in the HFD-fed ASKO mice. Notably, the down-regulation of lipolysis-related genes and the decreased response to CL-316,243 stimulation in the HFD-fed ASKO mice suggested that these animals exhibited impaired lipolysis. In addition, the HFD-fed ASKO mice displayed impaired energy expenditure, indicating that the miR-200b/a/429 cluster is essential for developing adaptive responses to stressors such as HFD. For the first time, our studies demonstrated the essential role of miR-200b/a/429 in adipocytes in the regulation of HFD-induced whole-body metabolic changes.

## INTRODUCTION

Adipose tissue is a fat storage depot and an endocrine organ that regulates whole-body fatty acid homeostasis [1]. There are two functionally distinct adipose tissues in mammals, white adipose tissue (WAT) and brown adipose tissue (BAT) [2], and the location, morphology and function are distinct for WAT and BAT. Adipose tissue dysfunction is the primary cause of obesity and obesity-related metabolic disorders such as insulin resistance, diabetes and cardiovascular disease [3].

The enrichment of miR-1, miR-206 and miR-133 in BAT suggests distinct miRs profiles between WAT and BAT [4]. Robust evidence has demonstrated the role of miRs in adipogenesis, adipocyte differentiation [5, 6] and in the browning of white adipocytes [7, 8]. Growing evidence shows that miRs are involved in obesity and obesity-related metabolic disorders [9], such as miR-223 [10], miR-378 [11], and miR-103/107 [12].

The highly conserved miR-200 family is composed of five members, miR-200a, miR-200b, miR-200c, miR-141 and miR-429, which are similar in sequence and are clustered and expressed as two separate polycistronic pri-miR transcripts: the miR-200b/a/429 cluster on chromosome 4 and the miR-200c/141 cluster on chromosome 6. The miR-200 family has been reported to be strongly expressed in lung, kidney and thymus tissues [13]. Previous studies showed the critical functions of miR-200 family in female reproduction [14, 15]. Hasuwa et al revealed the requirement of miR-200 family and their target zeb1 in hypothalamo-pituitary-ovarian axis to support female ovulation [13]. The miR-200 family has also been shown to be involved in the epithelial-to-mesenchymal transition in humans, thereby enhancing cancer cell colonization in distant tissues by targeting zeb1, and in the regulation of olfactory neurogenesis and osmotic stress in zebrafish [16, 17]. miR-8, the homolog of miR-200 in Drosophila, has been reported to positively regulate body size [18]. Recent studies have provided evidence that miR-200b has a role in beta cell apoptosis *in vitro* and *in vivo* [19, 20]. MiR-200, down-regulated in the livers of db/db mice, also contribute to IL-6-induced insulin resistance [21]. In hypothalamus, miR-200a inhibition of ob/ob mice reduced body weight and improved whole-body insulin sensitivity [22]. However, the role of the miR-200b/a/429 cluster in adipose tissue is currently poorly understood.

To study the function of miR-200b/a/429 in mammalian energy homeostasis, we generated adipose tissue-specific miR-200b/a/429 knockout (ASKO) mice and revealed the critical role of this miR in modulating lipolysis and energy expenditure, consequently regulating obesity during periods of dietary stress. Thus, the miR-200b/a/429 cluster may serve as a potential target for therapeutic intervention in obesity and metabolic syndrome.

## RESULTS

### Expression pattern of miR-200b/a/429 in adipose tissue and generation of the ASKO mice

**Figure 1:**
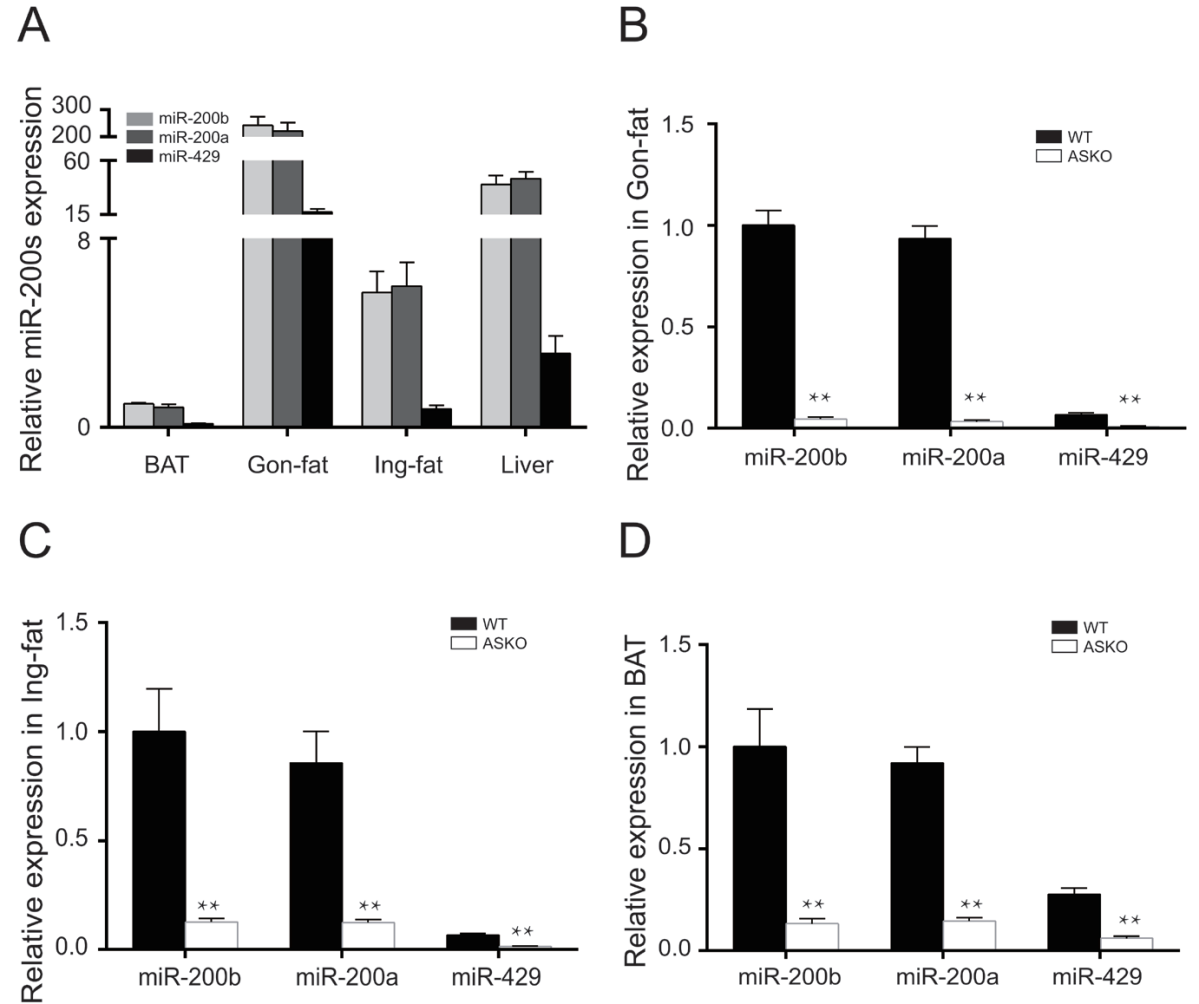
Expression pattern of miR-200b/a/429 in adipose tissue. A. Relative miR-200b/a/429 expression in BAT, Gon-fat, Ing-fat of WT mice (*n* = 4). Light gray bars, miR-200b; dark gray bars, miR-200a; black bars, miR-429. **B.**-**D.** MiR-200b/a/429 expression in the Gon-fat **B.**, the Ing-fat **C.** and the BAT **D.** of 15-week-old ASKO and WT mice (*n* = 6). All of the results are presented as the means ± SEM; *p < 0.05 and **p < 0.01.

To detect the miR-200b/a/429 expression levels in metabolic tissues, the abundance of miR-200b/a/429 in inguinal fat (Ing-fat), gonadal fat (Gon-fat), BAT and liver were measured by real-time PCR. Our data showed that the miR-200b/a/429 cluster was most strongly expressed in Gon-fat and least strongly expressed in BAT (Figure 1A).

The generation of a floxed miR-200b/a/429 mouse model was described in the Supplemental Methods, and the targeting construct was showed in Supplemental Figure 1A. To obtain mice in which miR-200b/a/429 cluster deletion was restricted to adipose tissue, the miR^flox/flox^ mice were crossed with transgenic mice expressing Cre recombinase driven by the Fabp4/aP2 promoter (Supplemental Figure 1A). Genomic DNA was extracted from tails and PCR was performed using specific primers to determine the genotypes (Supplemental Figure 1B). To ascertain the miR-200b/a/429 allele status, adipose tissue was collected and the expression level of miR-200b/a/429 was detected. Our results showed that the Cre^+^/miR^flox/flox^ mice displayed significantly lower expression levels of miR-200b/a/429 in gonadal fat (Gon-fat, Figure 1B), inguinal fat (Ing-fat, Figure 1C), and BAT (Figure 1D) than the control (Cre^−^/miR^flox/flox^) littermates (hereafter designated as WT). The abundance of miR-200b/a/429 in liver, kidney and lung tissues was not altered (Supplemental Figure 1C-1E). The ASKO mice were born at the expected Mendelian frequencies and were indistinguishable from the WT littermates.

### The HFD-fed ASKO mice exhibited increased body weight and adiposity

**Figure 2:**
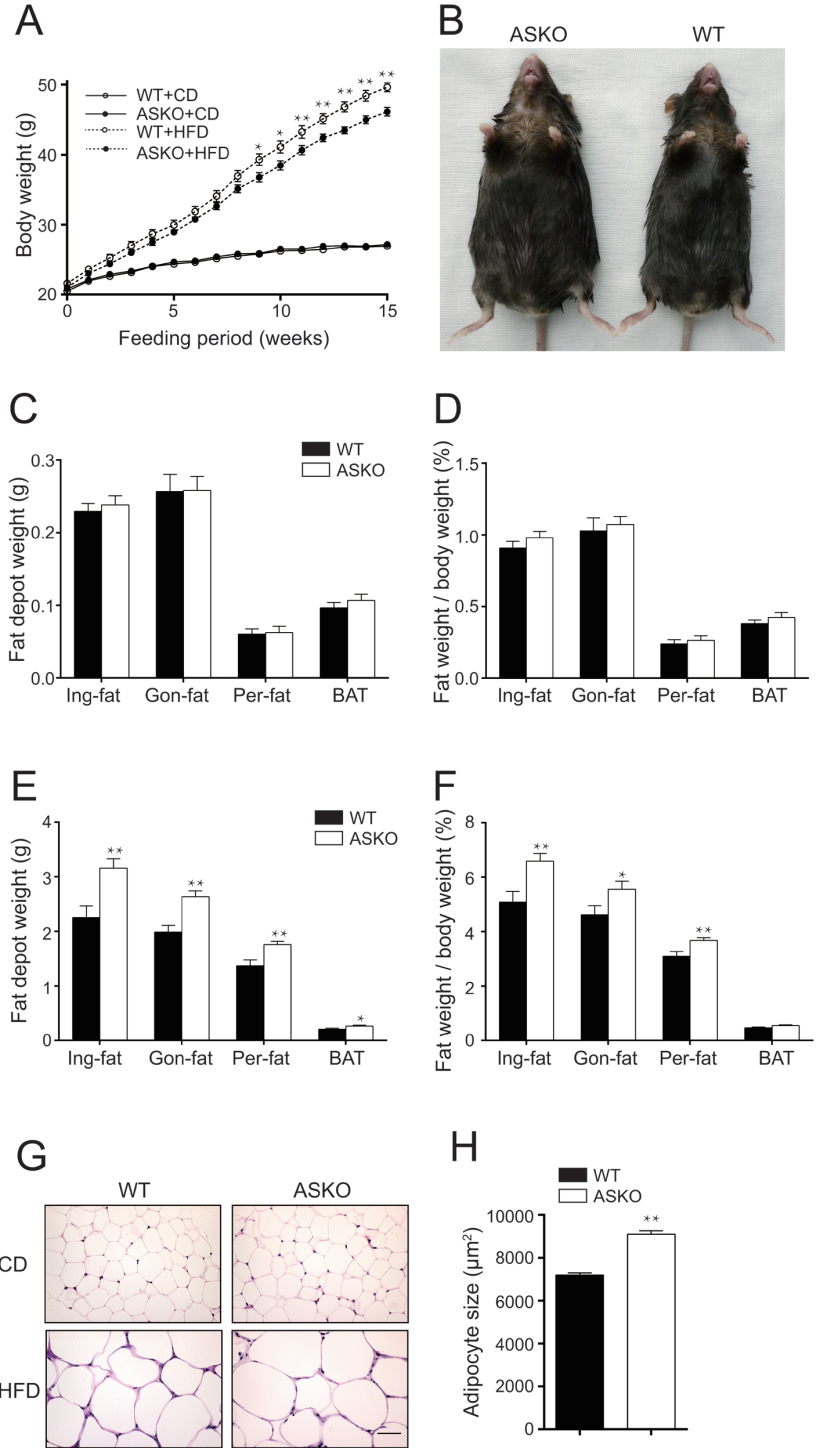
Increased body weight and fat content in ASKO mice under HFD conditions. A. Body weight of the WT and ASKO mice on both diet conditions (*n* > 10). **B.** Representative photographs of mice exhibiting HFD-induced obesity. **C.**-**D.** Organ weight analysis from CD-fed **D.**-**F.**, *n* = 12) and HFD-fed **E.**-**F.**, *n* > 11) mice, including absolute fat pad weight, the percentage of the total body weight and absolute weight of other peripheral organs, as indicated in the figure. **G.** H&E staining of the Gon-fat from ASKO and WT mice under both diets conditions. Scale bar, 50 μm. **H.** The mean adipocyte area in the gonadal fat (square micrometers) of the HFD-fed mice. The values represent the means ± SEM. *p < 0.05; **p < 0.01.

As shown in Figure 2A, the ASKO mice displayed a similar growth rate to the WT mice when fed the CD (Figure 2A). However, when fed the HFD for 15 weeks, the ASKO mice exhibited an increased body weight, and the difference reached significance after 9 weeks of HFD induction (Figure 2A). Figure 2B shows a representative photograph of the ASKO and WT mice, and the ASKO mice weighed approximately 9.2% more than the WT mice after 15 weeks of HFD feeding (Supplemental Figure 2A).

Next, to elucidate the underlying basis for this increase in body weight, we weighed inguinal Ing-fat, Gon-fat), perinephric fat (Per-fat), BAT and other organs from the ASKO and WT mice under both diet conditions. Our data revealed no difference in the weight of any fat tissue (Figure 2C-2D) or other peripheral organ (Supplemental Figure 2B) under CD conditions between the two genotypes mice. In contrast, all fat pads from the HFD-fed ASKO mice weighed more than the fat pads from the WT mice (Figure 2E-2F). All fat pads were collected together and weighted under HFD condition. Compared to the total weight of adipose tissue from WT mice, those from the ASKO mice are significantly increased with 34.6%. No difference was observed in the weight of other peripheral organs under HFD conditions (Supplemental Figure 2C). Taken together, our data suggested that miR-200b/a/429 deletion from adipocytes leads to a higher body weight due to increased adiposity under HFD conditions.

Furthermore, to determine whether the increased amount of fat in ASKO mice is due to larger fat cells, the histomorphology of the adipose cells from Gon-fat and BAT were analyzed. HE staining of the Gon-fat revealed the presence of enlarged adipocytes in the HFD-fed ASKO mice, whereas no difference in the adipocyte size was observed in the CD-fed mice (Figure 2G). This observation was further confirmed by a 26.5% increase in the average area of individual white adipocytes from the HFD-fed ASKO mice (Figure 2H). Interestingly, adipocyte hypertrophy was not observed in the BAT from the HFD-fed ASKO mice (Supplemental Figure 2D), suggesting a weak response of the BAT to HFD induction. These results indicated that the adipose tissue expansion observed in the ASKO mice is due to adipocyte hypertrophy.

### HFD-fed ASKO mice exhibit impaired insulin sensitivity

**Figure 3:**
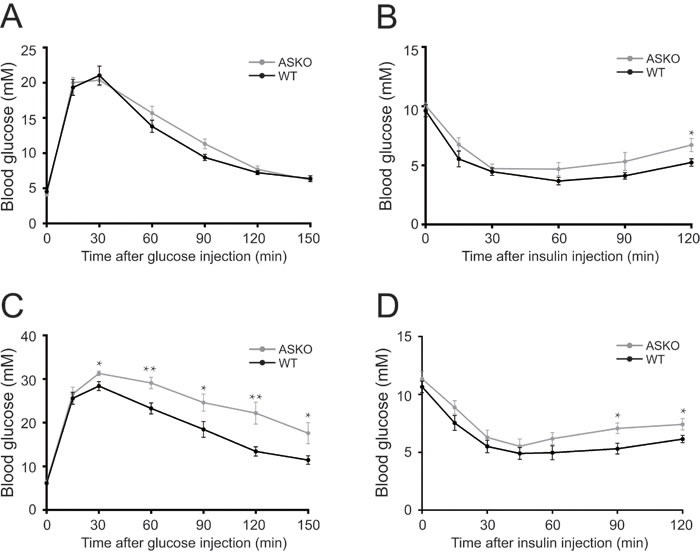
Impaired glucose tolerance and insulin sensitivity in the HFD-fed ASKO mice Blood glucose levels during IPGTT **A.**, **C.** and IPITT **B.**, **D.** in fasted WT (black) and ASKO (gray) under CD **A.**, **B.** and HFD **C.**, **D.** conditions (*n* = 7-10). The values represent the means ± SEM. *p < 0.05; **p < 0.01.

To test whether the increased fat weight alters insulin sensitivity in mutant mice, glucose metabolism was assessed under both diet conditions. As shown in Figure 3A and 3B, the response of blood glucose to exogenous glucose and insulin administration were quite similar between the CD-fed WT and ASKO mice (Figure 3A-3B). In contrast, under HFD conditions, marked phenotypic changes in glucose and insulin homeostasis were observed in the ASKO mice. The GTT assay suggested that the HFD-fed ASKO mice exhibited glucose intolerance (Figure 3C), and the significantly elevated blood glucose content upon insulin administration indicated decreased insulin sensitivity in the HFD-fed ASKO mice (Figure 3D).

Interestingly, the fasting insulin levels were significantly lower in the ASKO mice than in the WT mice under both CD and HFD conditions (Table 1). Additionally, decreased plasma adiponectin levels were observed in the ASKO mice compared to the WT mice, although this difference did not reach significance (Table 1). Taken together, these results strongly suggest that reduced adipose tissue insulin sensitivity results from decreased insulin levels and insulin sensitivity.

**Table 1:** Metabolic parameters of WT and ASKO mice fed a chow and high-fat diet

	CD		HFD	
	WT	ASKO	WT	ASKO
Plasma triglycerides (mg/dl)	47.20±3.33	51.50±3.72	79.54±3.38	102.53±9.64*
Plasma cholesterol (mg/dl)	69.44±5.32	59.00±3.22	129.68±5.33	108.27±4.84**
Plasma NEFA (mEq/L)	0.89±0.08	0.88±0.09	0.66±0.05	0.59±0.02
Plasma glycerol (mmol/L)	349.68±12.34	343.65±22.93	365.67±11.83	362.56±18.70
Plasma leptin (ng/ml)	1.74±0.20	1.71±0.14	40.74±2.49	44.57±3.37
Plasma adiponectin (μg/L)	18.75±2.09	16.95±2.52	14.42±0.93	13.17±1.73
Plasma insulin (ng/ml)	0.57±0.10	0.30±0.02*	3.80±0.78	1.81±0.51*

Data are expressed as mean±SEM. 16 hours fasting

* *P* < 0.05

** *P* < 0.01. *n*=7-8 mice.

### Lipolysis is impaired in the HFD-fed ASKO mice

**Figure 4:**
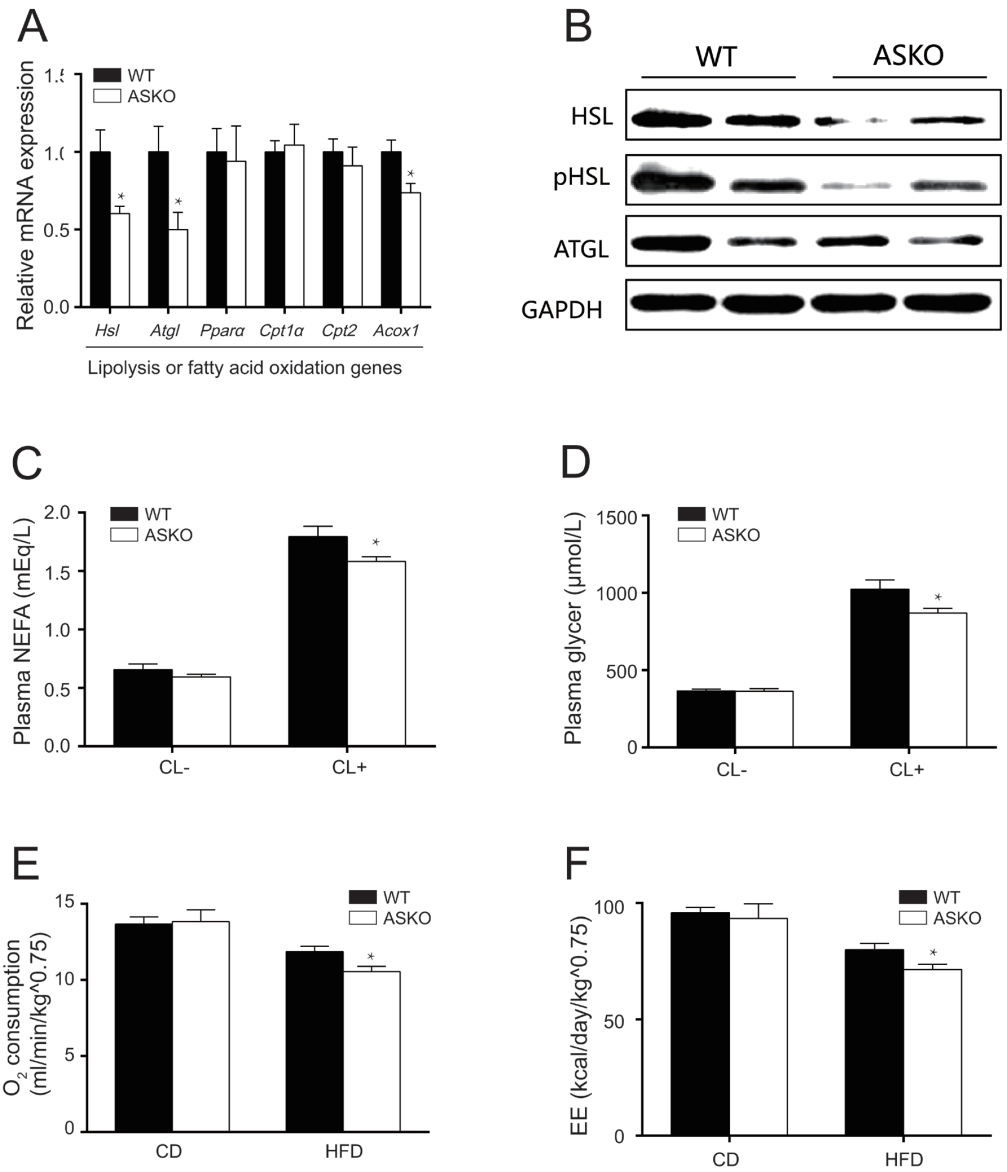
Ablation of miR-200b/a/429 reduces lipolysis and energy expenditure *in vivo* **A.** The mRNA expression of lipolysis-related genes in Gon-fat from HFD-fed WT and ASKO mice (*n* = 6). **B.** Representative immunoblotting for HSL, pHSL, ATGL in Gon-fat from HFD-fed mice. **C.**, **D.** The circulating levels of NEFA **C.** and glycerol **D.** on β3-AR agonist CL-316, 243 stimulation (*n* = 5-8). **E.** The O2 consumption and energy expenditure (EE) **F.** of male WT and ASKO mice was monitored during feeding with the CD or the HFD (*n* = 5-7). The values represent the means ± SEM. *p < 0.05; **p < 0.01.

To elucidate the potential molecular changes in adipose tissue that could account for the observed phenotypes, we examined the expression of key genes that have been reported to be involved in lipogenesis and lipolysis. We found that the lipogenesis genes were not altered (Supplemental Figure 3A), suggesting that the ASKO and WT mice displayed a comparable capacity for lipid biosynthesis. However, significantly decreased levels of the *Hsl* and *Atgl* were observed in the WAT from the HFD-fed ASKO mice (Figure 4A), and the differences were further confirmed by immunoblotting (Figure 4B). Acox1 (Acyl-coenzyme A oxidase 1), the first enzyme in peroxisomal fatty acid β-oxidation, was decreased in the HFD-fed ASKO mice (Figure 4A). These data led us to speculate that lipolysis is impaired in these HFD-fed ASKO mice.

Next, we examined whether lipolysis is altered *in vivo* in the WAT of HFD-fed ASKO mice following stimulation with the β3-adrenergic agonist CL-316,243. Our data showed that baseline NEFA and glycerol release was not affected by miR-200b/a/429 deficiency in adipose tissue (Figure 4C and 4D). Upon stimulation, although both the WT and ASKO mice exhibited robust lipolytic responses, significantly attenuated lipolysis was observed in the HFD-fed ASKO mice (Figure 4C and 4D).

Consistent with the reduced lipolysis in the HFD-fed ASKO mice, the plasma TAG levels were significantly higher in these mice (*p* < 0.05) (Table 1). Surprisingly, no significant differences in the plasma NEFA or glycerol levels were observed (Table 1). However, ASKO mice exhibited significantly lower total cholesterol levels under both diet conditions. Taken together, our data suggested that miR-200b/a/429 deficiency in adipose tissue results in reduced lipolysis, leading to a progressive increase in body weight in the ASKO mice under HFD conditions.

### Energy expenditureare are impaired in the ASKO mice

To determine whether the phenotypes observed in the HFD-fed ASKO mice are related to whole-body energy consumption, the mutant and WT mice were subjected to metabolic cage studies under both diet conditions. As shown in Figure 4E-4F and Supplemental Figure 4, under CD conditions, no significant differences in the metabolic parameters of the ASKO mice were observed. In contrast, metabolic phenotyping revealed decreased energy expenditure in the HFD-fed ASKO mice, as reduced oxygen consumption (Figure 4E) and energy expenditure (Figure 4F), without any change in food intake or physiological activity (Supplemental Figure 4A-4B). A non-significant decrease in CO_2_ production was observed in the HFD-fed ASKO mice (Supplemental Figure 4C). The RQ, which is the ratio of the amount of CO_2_ produced to oxygen consumed by breathing, was highly similar between the WT and ASKO mice under both diet conditions (Supplemental Figure 4D).

As BAT is a critical organ for energy homeostasis, we also measured the expression levels of genes known to be involved in BAT metabolism in the HFD-fed mice. Our data showed that the mRNA levels of these genes were not significantly altered in the HFD-fed ASKO mice (Supplemental Figure 3B).

### Putative metabolic targets of miR-200b

**Figure 5:**
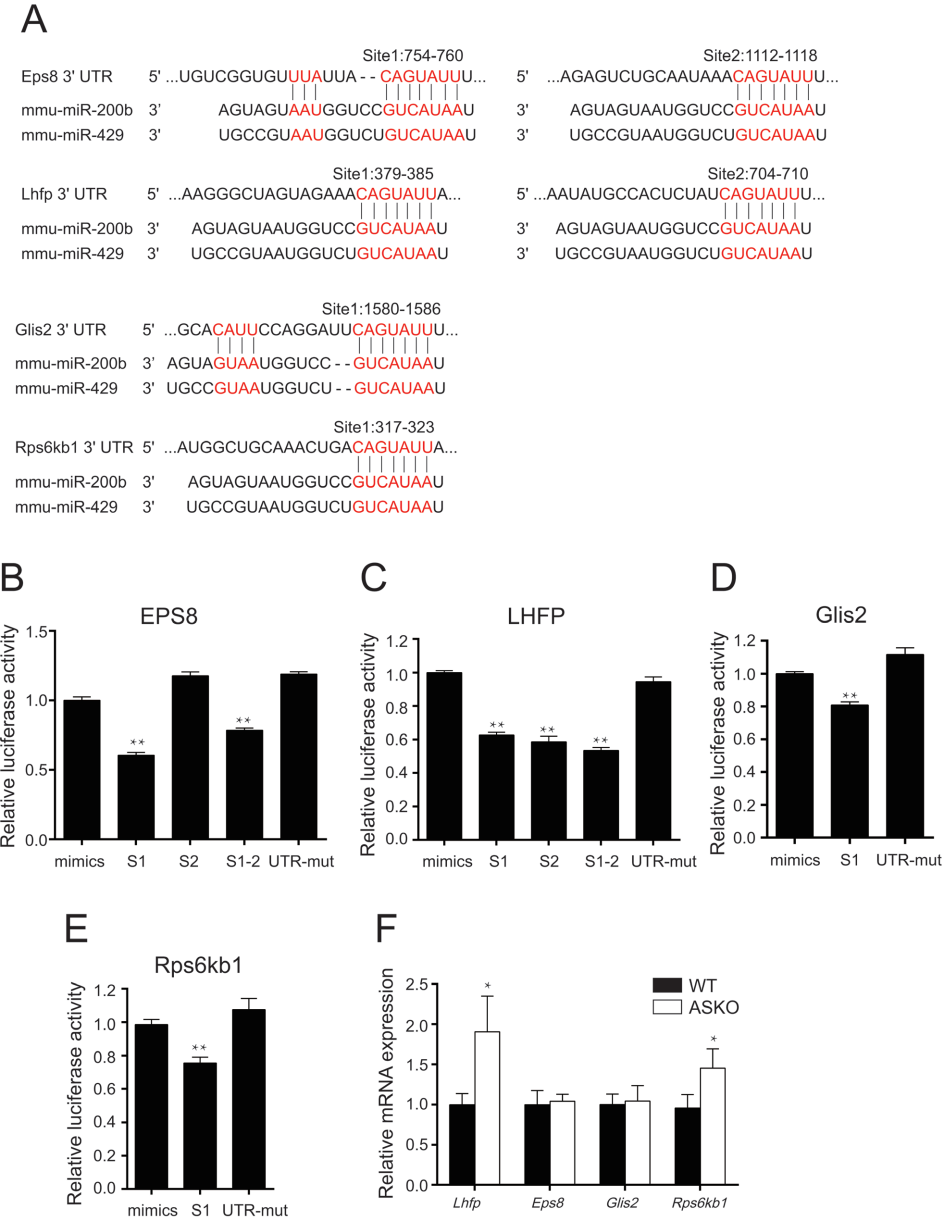
miR-200b targets the 3′UTRs of *Eps8, Lhfp, Glis2* and *Rps6kb1* **A.** TargetScan identified the conserved predicted miR-200b binding sites on the 3′UTRs of *Eps8*, *Lhfp*, Glis2 and *Rps6kb1*. **B.**-**E.** Quantification of the dual-luciferase assay in HEK293 cells co-transfected with an miR-200 mimic or control oligonucleotides and the *Eps8* **B.**, *Lhfp* **C.**, *Glis2* **D.**, *Rps6kb1* **E.** or mutant 3′UTR reporter plasmid (*n* = 4). **F.** mRNA expression of *Eps8*, *Lhfp*, *Glis2* and *Rps6kb1* genes in Gon-fat of the WT (black bars) and ASKO (white bars) mice (*n* = 6). mut, mutant. The values are expressed as the means ± SEM. *p < 0.05, **p < 0.01.

The TargetScan prediction program was used to identify target genes containing binding sites for the miR paralog miR-200b. Four predicted target genes, epidermal growth factor receptor pathway substrate 8 (*Eps8*), lipoma HMGIC fusion partner (*Lhfp*), GLIS family zinc finger 2 (*Glis2*) and ribosomal protein S6 kinase, polypeptide 1 (*Rps6kb1*), which have been reported to be predominately associated with obesity and insulin resistance, were selected for further validation *via* luciferase reporter assays. The predicted miR-200b binding sites on the 3′UTRs of those four genes are shown in Figure 5A. Luciferase assays revealed the repression of the *Eps8* 3′UTR S1 reporter by a miR-200b mimic, whereas this repressive effect was diminished for the *Eps8* 3′UTR S2 reporter and mutated reporter (Figure 5B). As shown in Figure 5C-5E, the luciferase activities were reduced in the cells transfected with a plasmid containing the 3′UTR of *Lhfp*, *Glis2* or *Rps6kb1* carrying miR-200b binding sites in the presence of miR-200b mimics, whereas these inhibitory effects were not observed for the non-specific scrambled oligonucleotides (Figure 5C-5E). Furthermore, real-time PCR data revealed the significant elevation of *Lhfp* and *Rps6kb1* in the HFD-fed ASKO mice. No difference in *Eps8* and *Glis2* RNA expression was observed between the ASKO and WT mice (Figure 5F), suggesting that *Eps8* and *Glis2* genes may be post-transcriptionally regulated by miR-200b/429.

## DISCUSSION

In this study, to explore the potential effect of miR-200b/a/429 in adipocytes on metabolic homeostasis, ASKO mice were successfully generated leaving the host gene Ttll10 intact (data not shown). The ASKO mice were born at the expected Mendelian frequencies and were indistinguishable from their WT littermates fed the CD. However, when fed the HFD, the ASKO mice developed diet-induced obesity, demonstrating that adipose miR-200b/a/429 does not positively regulate systemic growth of mice. It has been reported that Drosophila lacking miR-8, the sole homolog of miR-200b/a/429, exhibit a significantly smaller body size and reduced insulin signaling [18]. Our data suggest either that the role of the miR-200 family in the regulation of body weight homeostasis is not evolutionarily conserved or that mice are not completely analogous to flies. No elevation of liver fat deposition was observed based on H&E staining analysis (Supplemental Figure 3C), we speculate that fatty liver may ultimately develop in the ASKO mice over time for the high level of serum total triglycerides.

Similar to other mouse strains carrying a global deletion of miR-378/378* [11], our ASKO mice exhibited overt phenotypes in response to excessive calorie intake. Mice lacking miR-378/378* are lean and resistant to HFD-induced obesity, whereas our ASKO mice are sensitive to diet-induced obesity. Impaired lipolysis in the WAT was observed in the ASKO mice, as indicated by the significantly down-regulated expression levels of the lipolysis-related genes and the significantly alleviated alterations in the serum FA and glycerol levels upon CL-316,243 stimulation. Surprisingly, in the absence of stimulation, no changes in the serum FA and glycerol levels of the ASKO mice were observed under either diet condition. We speculated that this lack of an effect could be due to either less severe lipolysis defects or to a global deceleration of FA turnover in ASKO mice. This finding is in agreement with previous observations in many obese individuals [23] and HSL haploinsufficient mice [24]. We also observed the significant down-regulated Acox1 in the HFD-fed ASKO mice, which might decrease the utilization of fatty acids and ultimately lead to fat deposition. Taken together, all these observations could partially explain the HFD-induced obesity of ASKO mice and the detailed mechanisms still need to be further explored.

Furthermore, we found that disrupted adipose tissue homeostasis leads to glucose intolerance in the HFD-fed ASKO mice. Unexpectively, we observed the lower level of serum insulin content in ASKO mice, which might due to the dysfunction of pancreas and pancreatic islet. Taken together, our data suggested that the glucose intolerance of those mice might be caused by both low serum insulin levels and insulin resistance. Normally, the low level of serum insulin content is associated with better insulin sensitivity, the opposite observation in our study might due to the failure of the signaling pathways of IRS-PI3K-Akt, as miR-200b has been reported to be involved in this pathway [18, 25, 26]. In this regard, the precise role of the miR-200b/a/429 cluster on the signaling pathways of IRS-PI3K-Akt in the target organs of insulin resistance must be further explored. It has been established that many miRs exert regulatory effects on insulin signaling and glucose metabolism at multiple levels [9, 27, 28]. Trajkovski et al. overexpressed miR-107 predominantly in mouse adipose tissue *via* adenovirus injection and observed impaired glucose tolerance and insulin sensitivity [12]. It is of interest to investigate whether the adenoviral injection of miR-200b/a/429 into the adipose tissue of HFD-induced ASKO mice restores insulin signaling.

It is well known that obesity is generally caused by an imbalance between energy intake and energy expenditure [29, 30, 31]. In this study, metabolic phenotyping analysis revealed that the energy balance was altered in the HFD-fed ASKO mice. Consistent with our finding, impaired energy expenditure was observed in mice lacking miR-378/378* [11] and in miR-196 transgenic mice [32].

MiRs modulate complex physiological and pathological pathways *via* multiple target networks. The miR-200b/a/429 complex undoubtedly targets many genes. Its putative targets were predicted computationally, and luciferase reporter assays suggested that the miR-200b/429 cluster targets the eps8, lhfp, glis2 and rps6kb1 genes. According to the mutual exclusion model of miR-mRNA interactions, cells in which an miR is silenced should highly express its targets [33, 34, 35]. Thus, we hypothesized that these targets were up-regulated at the protein level in the WAT of ASKO mice. In this regard, our data are in agreement with the observation that mice carrying a genetic deletion of Eps8, a regulator of actin dynamics, leads to reduced body weights and partial resistance to diet-induced obesity [36]. Similarly, it has been reported that the absence of Rps6kb1, another putative miR-200b/a/429 target, in mice protects against age- and diet-induced obesity [33]. Taken together, we propose that the miR-200b/a/429 cluster exerts its regulatory functions *via* its targets, such as Eps8 and Rps6kb1. However, further investigation is required to elucidate the molecular mechanisms and regulatory pathways by which these miRs alter fat metabolism in HFD-fed ASKO mice.

In summary, our data reveal the role of adipose miR-200b/a/429 in HFD-induced obesity and insulin resistance, and suggest a promising strategy for addressing the health issues caused by obesity and its associated diseases.

## MATERIALS AND METHODS

### Animals

The mice were maintained in a C57BL/6 background, were housed in a pathogen-free facility at the Institute of Animal Science of the Chinese Academy of Agricultural Sciences (CAAS, Beijing, China) in a 12 h light-dark cycle. Mice were provided with free access to standard irradiated normal rodent chow diet (CD; diet 1035; HFK Bioscience, Beijing, China) for 22 weeks. All experiments involving mice were approved by the Institutional Animal Care Research Advisory Committee of the CAAS.

### High-fat-diet treatment

Once mice were genotyped, they were aliquot randomly into high-fat-diet (HFD; 60% of energy from fat; D12492; Research Diets, New Brunswick, NJ, USA) and chow diet groups. HFD was fed to 20 g WT and ASKO male mice and the body weight was measured and recorded weekly. After 15-week of HFD induction, ASKO and WT mice were used for the following experiments and assays, including adipose tissues histological stains, the blood parameters, GTT, ITT, metabolic cages analysis and Western blot.

### Real-time PCR analysis

Total RNA was isolated from mouse tissue using TRIzol (Invitrogen, Carlsbad, CA, USA) according to the manufacturer's instructions. Then, 2 μg of total RNA was reverse-transcribed using the First Strand cDNA Synthesis Kit (Thermo Scientific, Waltham, MA, USA). QRT-PCR was performed using SYBR Green master mix and the 7500 Fast Real Time PCR system (Applied Biosystems, Warrington, UK). The primers used for real-time PCR are shown in Supplemental Table 1. QRT-PCR of the miRs was performed using TaqMan MicroRNA assay kits for mmu-miR-200b-3p, mmu-miR-200a-3p, mmu-miR-429-3p and U6 snRNA (Applied Biosystems, Foster City, USA) according to the manufacturer's instructions. MiR expression analysis was conducted using TaqMan Universal PCR Master Mix (Applied Biosystems, Foster City, CA USA). Gene expression was normalized to U6 or *Gapdh* for miRs and mRNAs, respectively. Relative gene expression was calculated using the comparative cycle threshold (2^−ΔΔCt^) method [37].

### Western blotting

Tissues were lysed in T-PER Tissue Protein Extraction Reagent (Thermo-Fisher, Rockford, MD, USA) in the presence of a protease inhibitor cocktail (Roche, Shanghai, China). The lysates and protein markers (Fermentas, York, UK) were resolved *via* SDS-PAGE, transferred to PVDF membranes (Millipore, Bedford, MA, USA), and probed using anti-Hsl (cat. no. 4107, CST, Danvers, MA, USA), anti-phospho-HSL Ser563 (cat. no. 4139, CST, Danvers, MA, USA), anti-ATGL (cat. no. 2138, CST, Beverly, MA, USA) and anti-GAPDH antibodies (cat. no. 8884, CST, Beverly, MA, USA). The immunoreactive bands were detected using Pierce ECL Western Blotting Substrate (Thermo, Waltham, MA, USA). The protocol was described in our previous study [38].

### Metabolic analyses

The HFD-fed and CD-fed mice (*n* = 5-7) were housed in individual metabolic chambers and were allowed to acclimatize for 24 h before data collection. Energy expenditure of these mice was measured using an indirect open-circuit calorimeter (Oxylet, Panlab, Barcelona, Spain). Data on O_2_ consumption (VO_2_; ml/min/kg^0.75) and CO_2_ production (VCO_2_; ml/min/kg^0.75) were collected every 30 min. Locomotor activity was measured by continuously recording spontaneous activity using extensiometric weight transducers placed below the cages, which enabled the detection of activity even without displacement. The respiratory quotient (RQ) was calculated as the ratio of VO_2_ to VCO_2_, and energy expenditure was determined as (3.815 + 1.232 × RQ) × VO_2_ and was expressed as kcal/h/mouse [39]. The data is normalized to body weight.

### Glucose and insulin tolerance tests

We measured the blood glucose concentration using a glucose meter (OneTouch, Milpitas, CA, USA) for the intraperitoneal glucose tolerance test (IPGTT) and the intraperitoneal insulin tolerance test (IPITT) (*n* = 7-10). We performed the IPGTT after a 16-h fast using an i.p. injection of 2 g of glucose/kg body weight. For the IPITT, the fasting blood glucose level was measured (4-h fast, blood collected from the tail vein). Then, insulin was injected i.p. (0.75 U/kg body weight, Humulin, Eli Lilly, Indianapolis, IN, USA), and blood samples were collected immediately before (time 0) and at 30, 60, 90, 120 and 150 min after insulin injection for glucose measurement.

### Blood analysis

Blood was obtained *via* the caudal vein before the mice (*n* = 7-8) were sacrificed. The TAG and cholesterol contents were measured using enzymatic assay kits from Applygen (Beijing, China). The plasma insulin, leptin and adiponectin levels were measured *via* ELISA (Millipore, Bedford, MA, USA), and the free glycerol content (Sigma, St. Louis, MO, USA) and the nonesterified fatty acid (NEFA) levels were measured *via* a colorimetric assay (Wako Chemical, Osaka, Japan).

### Histological studies

The tissues from HFD- and CD- diet mice with different genotypes (*n* = 4) were dissected, fixed in 4% para­formaldehyde overnight and rinsed with phosphate-buffered saline, followed by embedding in paraffin. The paraffin-embedded WAT, BAT and liver tissues were sectioned at a thickness of 5 μm and stained with hematoxylin and eosin (H&E). The adipocyte size of Gon-fat from HFD-fed WT and ASKO mice were measured by using Image J software as previously described [40]. Adipocyte size was determined on sections and measured in five different sections from each mice fat pad (four mice per group), after which the area of 50 cells in each section were measured.

### Lipolysis

For *in vivo* lipolysis assay, HFD-fed WT and ASKO mice were fasted for 4 hours and injected i.p. with the β3-specific agonist CL-316,243 (CL, 0.1 mg/kg, Sigma, MO, USA). Blood was collected before and 5 min after injection for the measurement of the NEFA and glycerol levels.

### Luciferase reporter constructs and assay

The 3′UTR portions of the mouse *Eps8*, *Lhfp*, *Glis2* and *Rps6kb1* mRNAs were cloned into the psiCHECK-2 vector (Promega, Madison, WI, USA). The 3′UTR portions of target genes not corresponding to a target sequence were used as controls. The specific primers are listed in Supplemental Table 1. Then, 100 ng of each psiCHECK-2 construct was co-transfected with 50 nM miR mimics or a negative control into HEK293 cells in a 24-well plate using Lipofectamine 2000 (Invitrogen, Carlsbad, CA, USA). HEK293 cell lines were maintained in DMEM supplemented with 10% FBS at 37 °C in a 5% CO_2_ environment. After 48 hours, the cell extracts were obtained, and the firefly and Renilla luciferase activity levels were measured using the Dual-Luciferase reporter system (Promega, Madison, WI, USA) according to the manufacturer's instructions.

### Statistical analysis

The data are presented as the means ± s.e.m. The values were analyzed *via* two-tailed independent sample Student's t tests, using GraphPad Prism version 6 (GraphPad Software, La Jolla, CA, USA). A value of *p* < 0.05 was considered to be significant.

## SUPPLEMENTARY MATERIALS FIGURES AND TABLE


