# Supplementary material for: Adipocyte miR-200b/a/429 ablation in mice leads to high-fat-diet-induced obesity
Source: Oncotarget. 2016 Sep 16;7(42):67796–807. doi: 10.18632/oncotarget.12080 (PMC5356520; doi:10.18632/oncotarget.12080)
Supplement: Supplementary file 1 [file oncotarget-07-67796-s001.pdf]

Adipocyte miR-200b/a/429 ablation in mice leads to high-fat-diet-induced obesity

Supplementary Material

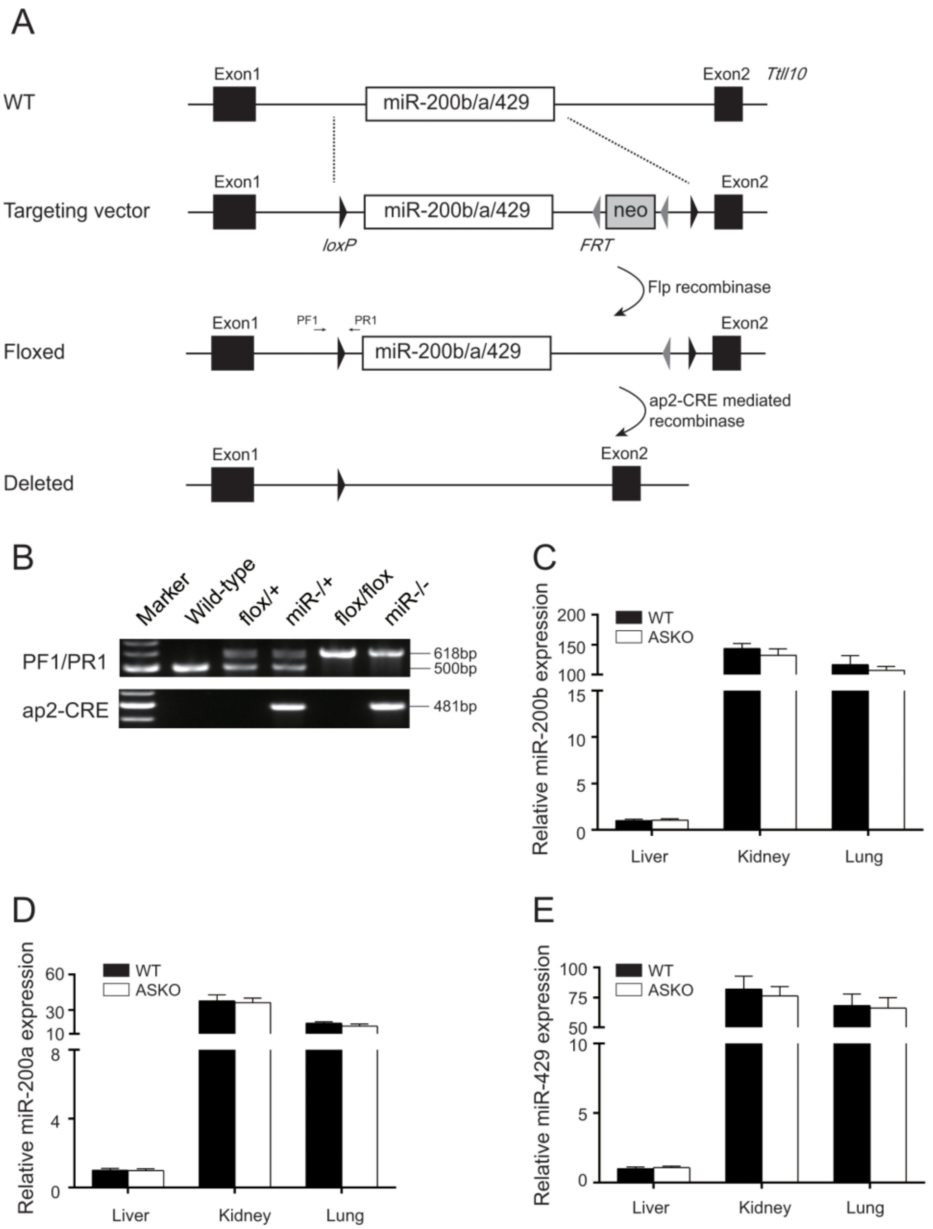

Supplementary Figure 1 Generation of the ASKO mice. (A) Schematic representation of targeting construction for the generation of floxed mice and the primers used for genotyping. (B) PCR genotyping for WT, heterozygotes and homozygotes. Mir-200b (C), mir-200a (D) and mir-429 (E) expression in the liver, kidney and lung of 15-week-old WT mice (n=6). All results are presented as the means ± SEM; $*p<0.05$ and $**p<0.01$.

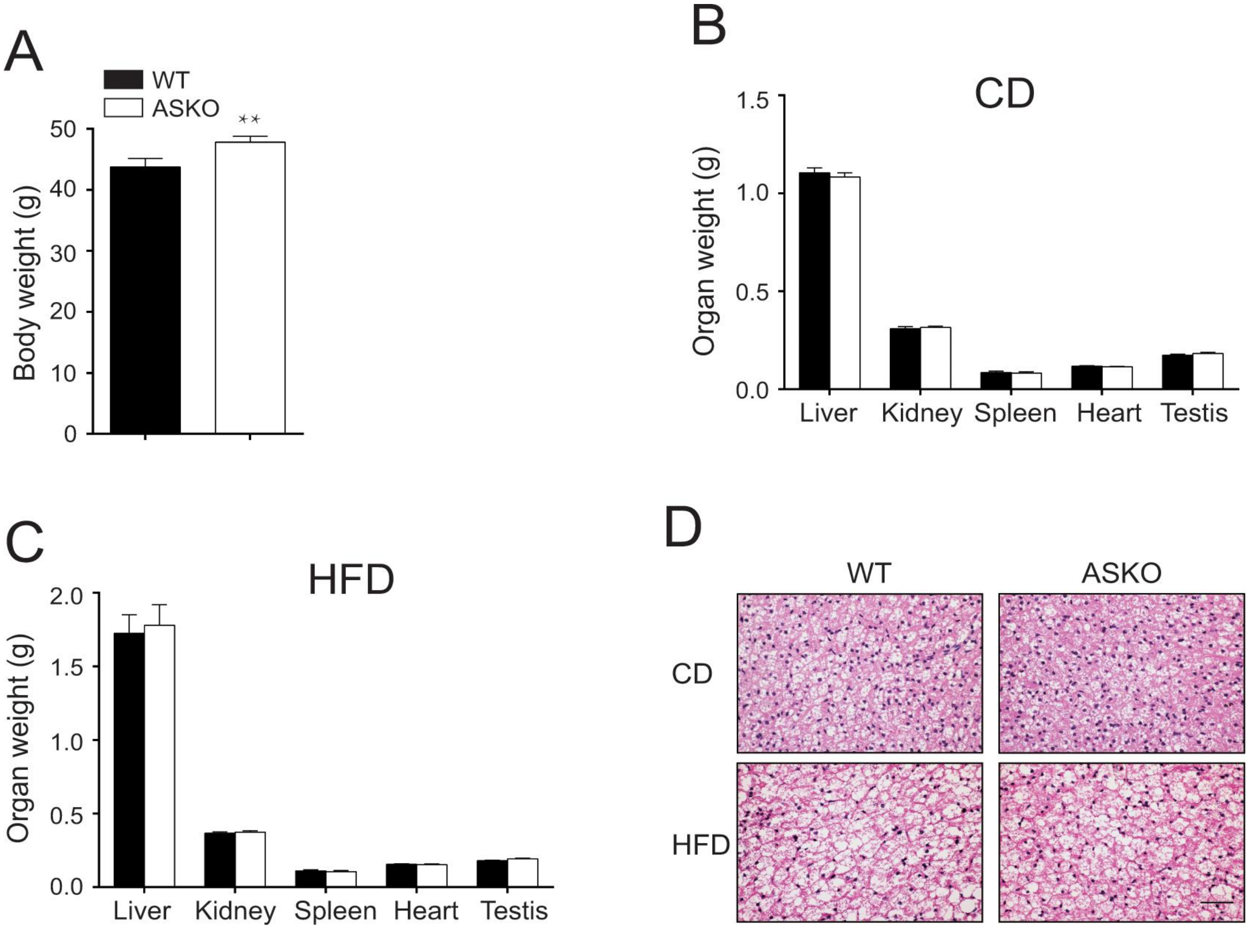

Supplementary Figure 2 (A) Comparison of body weights of the WT and ASKO mice after 15 weeks of HFD induction. (B-C) Organ weight analysis from CD-fed (B, n=12) and HFD-fed (C, n≥11) mice. (D) H&E staining of the BAT from ASKO and WT mice under both diets conditions. Scale bar, 50 μm.

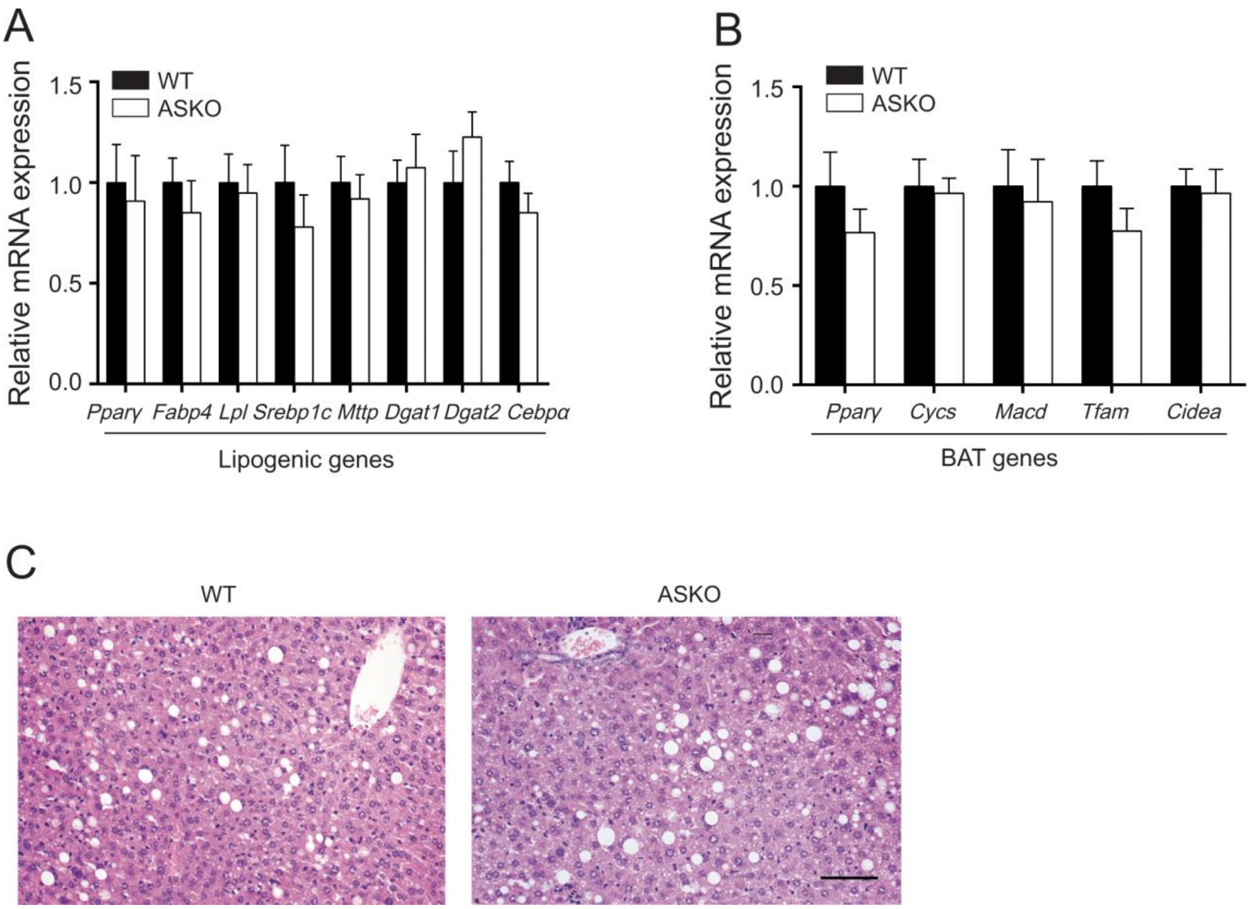

Supplementary Figure 3 Analysis of lipogenesis, brown adipogenesis and morphology in different tissues of ASKO and WT mice. Gene expression in WAT (A) and BAT (B) mRNA expression of genes involved in lipid metabolism in gonadal adipose tissue from HFD-fed mice (n=6). (C) Representative micrographs of H&E staining of liver sections from HFD-fed mice. Scale bar, 50 µm. All results are presented as the means ± SEM; *p<0.05 and **p<0.01.

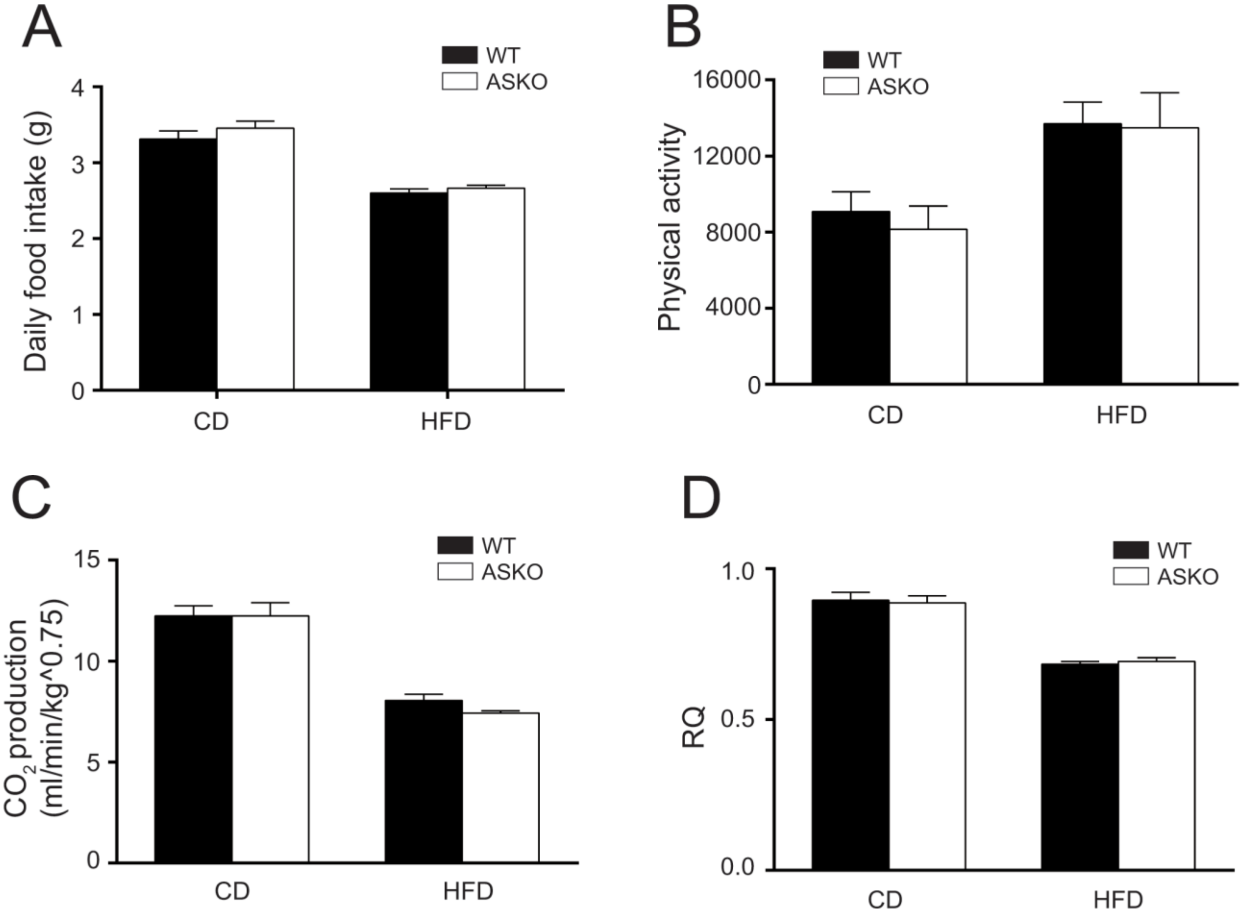

Supplementary Figure 4 Metabolic parameters were measured for WT and ASKO mice under both diet conditions (n=5-7), including the food intake (A), physical activity (B), the CO_2 production (C) and RQ (D). The values are expressed as the mean ± SEM. *$p<0.05$, **$p<0.01$.

Generation of the flox mice

To generate a floxed miR-200b/a/429 mouse model, a targeting construct was generated in which loxP sites flanked miR-200b/a/429 (Supplementary Figure S1a). A neomycin cassette flanked by FLP recombination sites was also inserted in the targeting construct. Recombinant embryonic stem cells in which the targeting vector was homologously integrated were microinjected into C57BL/6J mouse blastocysts. The resulting chimeras were mated to WT C57BL6 mice to generate F1 offspring carrying the recombinant miR-200b/a/429 allele. This work was done with the help of the Model Animal Research Center of Nanjing University. Then, the neomycin cassette was removed by cross-breeding the F1 mice with C57BL6 mice expressing FLP recombinase to generate males and females harboring the floxed miR-200b/a/429 allele (miRflox/flox). The ASKO mice were generated by breeding miRflox/flox mice with mice expressing Cre recombinase under the control of the Ap2 promoter (Jackson Laboratory, Bar Harbor, ME, USA). Genotyping was performed via PCR using the following primers: for the Cre transgene: 5’-GCCTGCATTACCGGTCGATGC-3’ and 5’-CAGGGTGTTATAAGCAATCCC-3’; for the upstream loxP site: 5’-ACAGGGCTTGAGCCACCTGT-3’ and 5’-CCAATGGCCAGGTCAGGATCAGG-3’; and for the downstream loxP site: 5’-AAATTGCATCGCATTGTCTG-3’ and 5’-ATTCTCACTCCAAGGCTGAC-3’.

Supplementary Table 1

Specific primers for qPCR:

Dgat-1 sense sequence: 5’-GGTGCCGTGACAGAGCAGAT-3’
antisense sequence: 5’-CAGTAAGGCCACAGCTGCTG-3’
SREBP1C sense sequence: 5’-GGAGCCATGGATTGCACATT-3’
antisense sequence: 5’-GCTTCCAGAGAGGAGGCCAG-3’
Cycs sense sequence: 5’-GAGTTTTGGGCTGATGGGTA-3’
antisense sequence: 5’-ATCCCGCTGTAACACCAGTC-3’
Mcad sense sequence: 5’-TTTCGAAGACGTCAGAGTGC-3’
antisense sequence: 5’-TGCGACTGTAGGTCTGGTTC-3’
Cpt1a sense sequence: 5’-CCAGGCTACAGTGGGACATT-3’
antisense sequence: 5’-GAACTTGCCCATGTCCTTGT-3’
ACOX1 sense sequence: 5’-CAGGAAGAGCAAGGAAGTGG-3’
antisense sequence: 5’-CCTTTCTGGCTGATCCCATA-3’
Cpt2-133bp sense sequence: 5’-TATGATGGCTGAGTGCTCCA-3’
antisense sequence: 5’-ATAGAGCTCAGGCAGGGTGA-3’
Dgat2 sense sequence: 5’-GCGCTACTTCCGAGACTACTT-3’
antisense sequence: 5’-GGGCCTTATGCCAGGAAACT-3’
Mttp sense sequence: 5’-CAAGCTCACGTACTCCACTGAAG-3’
antisense sequence: 5’-TCATCATCACCATCAGGATTCCT-3’
IL6 sense sequence: 5’-CAAAGCCAGAGTCCTTCAGAG-3’
antisense sequence: 5’-GCCACTCCTTCTGTGACTCC-3’
IL10 sense sequence: 5’-TGAATTCCCTGGGTGAGAAG-3’
antisense sequence: 5’-TCATTCATGGCCTTGTAGACAC-3’
Itgam sense sequence: 5’-CTGGTGCTCTTGGCTCTCAT-3’
antisense sequence: 5’-AAAAGGCCGTTACTGAGGTG-3’
PPARg sense sequence: 5’-CGGAAGCCCTTTGGTGACTT-3’
antisense sequence: 5’-CCTCGATGGGCTTCACGTTC-3’
TNFa sense sequence: 5’-AATGGCCTCCCTCTCATCAG-3’
antisense sequence: 5’-CCCTTGAAGAGAACCTGGGA-3’
HSL/lipe sense sequence: 5’-CCCTTCATGTCTCCTCTGCT-3’
antisense sequence: 5’-GCGGCAGATCTTCTACCACT-3’
LHFP sense sequence: 5’-GCTGGGACAGTGAGGAAGTC-3’
antisense sequence: 5’-GCCATCCATGTACACAGCAG-3’
GLIS2 sense sequence: 5’-GGAGAGGTGTAAAGCCTTGG-3’
antisense sequence: 5’-GCGATATTGGTGTTGTGTCG-3’
EPS8 sense sequence: 5’-GACGAACTGAGGTCTGTCTGC-3’
antisense sequence: 5’-CTGATCTTCTCCTGCCGTCT-3’
Rps6kb1 sense sequence: 5’-GCCCTGATGACTCCACTCTC-3’
antisense sequence: 5’-TCACCACTGCCTGGTCATAG-3’
PRDM16 sense sequence: 5’-TGAGCCCCAAGGAGTCTATG-3’
antisense sequence: 5’-GTCGGCTCCAAAGCTAACAG-3’
FABP4 sense sequence: 5’-TCACCTGGAAGACAGCTCCT-3’
antisense sequence: 5’-AATCCCCATTTACGCTGATG-3’
Tfam sense sequence: 5’-CACCCAGATGCAAAACTTTCAG-3’
antisense sequence: 5’-CTGCTCTTTATACTTGCTCACAG-3’
UCP1 sense sequence: 5’-GTGAAGGTCAGAATGCAAGC-3’
antisense sequence: 5’-AGGGCCCCCTTCATGAGGTC-3’
Cidea sense sequence: 5’-TGCTCTTCTGTATCGCCCAGT-3’
antisense sequence: 5’-GCCGTGTTAAGGAATCTGCTG-3’
Pgc1α sense sequence: 5’-CCCTGCCATTGTTAAGACC-3’

antisense sequence: 5’-TGCTGCTGTTCCTGTTTTC-3’
GAPDH sense sequence: 5’- TGCGACTTCAACAGCAACTC-3’
antisense sequence: 5’-ATGTAGGCCATGAGGTCCAC-3’

Specific primers for luciferase assay:
EPS8 3’UTR Sites 1&2:
Sense sequence: 5’-CCGCTCGAG AATCAAAGTAGCCCGTGTCG-3’
Antisense sequence: 5’-ATAAGAATGCGGCCGC ATTCTGTTCGACCCAAATGC-3’
EPS8 3’UTR Site 1:
Sense sequence: 5’-CCGCTCGAG AATCAAAGTAGCCCGTGTCG-3’
Antisense sequence: 5’-ATAAGAATGCGGCCGC TGCAGACTCTCTCCAGCTGAC-3’
EPS8 3’UTR Site 2:
Sense sequence: 5’-CCGCTCGAG TCAAAGAATATCGGGCCATC-3’
Antisense sequence: 5’-ATAAGAATGCGGCCGC ATTCTGTTCGACCCAAATGC-3’
EPS8 3’UTR mutant:
Sense sequence: 5’-CCGCTCGAG TCAAAGAATATCGGGCCATC-3’
Antisense sequence: 5’-ATAAGAATGCGGCCGC TGCAGACTCTCTCCAGCTGAC-3’
